# The relationship between childhood obesity and male puberty development: a systematic review and meta-analysis

**DOI:** 10.3389/fendo.2025.1711557

**Published:** 2025-11-27

**Authors:** Wenhua Feng, Chaonan Ren

**Affiliations:** Department of Pediatrics, Mianzhu People’s Hospital, Mianzhu, China

**Keywords:** boys, childhood obesity, puberty development, testicular enlargement, pubic hair development, peak height velocity

## Abstract

**Objective:**

This meta-analysis aimed to investigate the risks and physiological development differences during puberty in overweight and obese boys.

**Methods:**

Following the PRISMA 2020 guidelines and AMSTAR criteria, we systematically searched PubMed, EMBASE, and Web of Science, with a cutoff date of July 2025. After applying strict inclusion and exclusion criteria and performing quality assessments, statistical analysis was conducted using STATA 17.0 and Review Manager (version 5.4).

**Results:**

A total of 12 prospective cohort studies meeting the inclusion and exclusion criteria were included in the analysis. The meta-analysis indicated that overweight boys had a significantly higher risk of early testicular enlargement compared to normal-weight boys (overweight RR 1.38, obese RR 1.43). Regional differences were observed, with significant variations in the association between overweight status and early testicular enlargement risk in the U.S. and Asia-Pacific regions. Overweight and obese boys older than 6 years had a stronger association with early testicular enlargement. Furthermore, overweight boys experienced an earlier onset of early testicular enlargement compared to normal-weight boys (MD -0.23, p=0.002), while no significant difference was observed in obese boys (MD -0.23, p=0.10). In terms of pubic hair development, overweight (RR 1.24, p<0.00001) and obese boys (RR 1.42, p<0.00001) were at a higher risk for early development, and overweight boys developed pubic hair earlier (MD -0.38, p=0.0008). No significant difference was found in peak height velocity between obese and normal-weight boys (MD -0.32, p=0.09).

**Conclusion:**

Overweight and obesity have notable effects on male puberty development, particularly with respect to early testicular enlargement and pubic hair development.

**Systematic Review Registration:**

https://www.crd.york.ac.uk/prospero/, identifier, CRD420251172699.

## 1 Introduction

Puberty is a crucial transitional period in children’s growth, marking the shift from childhood to adulthood, characterized by rapid physical growth and significant development of secondary sexual characteristics (1, 2). In boys, key milestones of puberty include testicular development, the appearance of pubic hair, peak height velocity, voice changes, and the first ejaculation, all commonly used to assess the progress of puberty. The development of puberty is precisely regulated by the hypothalamic-pituitary-gonadal axis, which involves various sex hormones and metabolic factors (1, 3). Puberty in boys typically begins around age 9 and continues until around age 14. Early or delayed puberty development is closely associated with poor health and psychological distress (4). While the timing of puberty is influenced to some extent by genetic factors, nutrition and environmental factors also play significant roles (5).

Childhood overweight and obesity are global public health concerns, prevalent in both developing and developed countries. In China, it has been reported that up to 19.2% of children aged 7–18 are overweight or obese (6). The obesity rate among children aged 6 to 11 rose from 7% in 1980 to nearly 18% in 2012, while the proportion of obese individuals aged 12–19 increased from 5% to nearly 21% (7). Studies have consistently shown a clear association between childhood overweight and obesity and the early onset of puberty in girls. A growing number of longitudinal studies confirm that overweight or obese girls enter puberty earlier than their normal-weight counterparts, a trend observed globally. Obesity may influence puberty development through mechanisms such as activation of the hypothalamic-pituitary-gonadal axis, alterations in sex hormone levels, and metabolic disruptions (8). However, the relationship between childhood obesity and puberty development in boys remains inconsistent. Some studies suggest that obese boys experience earlier puberty onset than normal-weight boys, while others indicate a delay in puberty development in obese boys, even compared to normal-weight boys. Some research suggests that obesity may disrupt puberty development in boys by altering metabolic and endocrine factors, leading to changes in sex hormone secretion and delayed physiological development (9).

Existing studies on childhood obesity and puberty development in boys are primarily cross-sectional, which allows for the identification of correlations between obesity and puberty development but does not clarify causal relationships or explain underlying mechanisms and long-term effects. Additionally, the milestones used to assess puberty development vary across studies. For instance, some studies focus solely on testicular development and height growth, while neglecting other key factors that may influence puberty, such as voice changes and the occurrence of nocturnal emissions. This contributes to inconsistencies in results across different studies (10). Puberty is a multi-stage process that involves not only physical changes but also psychological, social, and physiological factors. Although some reviews and meta-analyses have discussed the impact of childhood obesity on puberty development, most of them focus exclusively on girls’ puberty development, either overlooking studies on boys or limiting the discussion to a single marker of precocity (11–13). Therefore, it is crucial to explore the comprehensive impact of childhood obesity on puberty development in boys. To gain a deeper understanding of how obesity affects puberty in boys, this study aims to conduct a systematic review and meta-analysis specifically examining the relationship between childhood obesity and puberty development in boys. Our findings may provide valuable insights into the risks of precocious puberty in overweight or obese boys.

## 2 Materials and methods

This evidence-based analysis follows the Preferred Reporting Items for Systematic Reviews and Meta-Analyses (PRISMA) 2020 guidelines and adheres to the Systematic Review Methodology Quality Assessment (AMSTAR) standards to ensure methodological rigor and high-quality analysis (14, 15). Overweight and obesity are defined according to the World Health Organization (WHO) criteria: overweight is a body mass index (BMI) between the 85th and 95th percentiles for children of the same age and sex, while obesity is defined as a BMI exceeding the 95th percentile. Tanner staging is an internationally recognized system for assessing the progress of puberty. Tanner Stage 2 marks the onset of puberty, characterized by an increase in testicular volume to 4 milliliters or greater and the appearance and spread of pubic hair. During puberty, boys also experience rapid height growth, the first ejaculation, and voice changes. These indicators are the focus of this review. Our systematic review has been prospectively registered on PROSPERO (CRD420251172699).

### 2.1 Literature search strategy

We conducted a comprehensive literature search in PubMed, Embase, and Web of Science, focusing on studies published from the earliest available records up to July 2025. These studies compared the growth and development of obese boys with that of non-obese boys. The following English keywords were used for the search: “boys,” “men,” combined with terms related to obesity, such as “overweight,” “obesity,” “obese,” “abdominal obesity,” and “central obesity,” as well as puberty-related terms, such as “puberty,” “precocious,” “gonadal development,” “pubic hair development,” and “peak height velocity.” For example, the specific search strategy in PubMed was: ((“Male” [Mesh] OR “boy” [Title/Abstract] OR “man” [Title/Abstract]) AND (“Overweight” [Mesh] OR “Obesity” [Mesh] OR “overweight” [Title/Abstract] OR “obese” [Title/Abstract] OR “obesity” [Title/Abstract] OR “abdominal obesity” [Title/Abstract] OR “central obesity” [Title/Abstract]) AND (“puberty” [Title/Abstract] OR “precocious” [Title/Abstract] OR “gonadarche” [Title/Abstract] OR “pubarche” [Title/Abstract] OR “Adolescent Peak Height Velocity” [Title/Abstract])). Additionally, we reviewed articles on childhood obesity and puberty development, including their reference lists, to ensure no relevant studies were overlooked.

### 2.2 Inclusion and exclusion criteria for literature

This study aims to compare puberty development in obese boys versus non-obese boys. Eligible studies will be included if they meet the following inclusion criteria:

Inclusion Criteria:

(1)Population: Studies involving children categorized as overweight/obese based on body mass index (BMI), waist circumference, or BMI trajectory (using data from at least two time points).
(2)Intervention: Studies that assess the impact of childhood overweight/obesity on puberty development, specifically the association with at least one puberty milestone in boys, including the onset of testicular development, appearance of pubic hair, and peak height velocity.
(3)Comparison: Studies comparing obese boys with non-obese boys.
(4)Outcome: The study must report at least one puberty development indicator for boys.
(5)Study Design: Cohort studies, case-control studies, or randomized controlled trials (RCTs).

Exclusion criteria:

(1)Studies where weight categories (normal weight, overweight, obese) are not clearly defined.
(2)Studies that do not include any puberty development indicators for boys.
(3)Studies that do not specifically report results for boys.
(4)Studies focusing on individuals with conditions that may affect sexual development, such as congenital gonadal developmental disorders, congenital adrenal hyperplasia, etc.

### 2.3 Assessment of the quality and evidence level of included studies

All studies included in this analysis are cohort studies. For the literature quality assessment, we used the Newcastle-Ottawa Scale (NOS) to evaluate the methodological quality of the studies (16). The scoring criteria of this scale include eight evaluation indicators: representativeness of the exposed cohort, selection of the unexposed cohort, exposure assessment, exclusion of individuals with relevant outcomes at the start of the study, comparability, outcome assessment, follow-up time, and adequacy of cohort follow-up. Each study is rated on a scale of 0 to 9, with the following quality classifications: low quality (0–3 points), moderate quality (4–6 points), and high quality (7–9 points). We assessed the risk of bias for each study based on these criteria. Additionally, we used the GRADE Pro 3.2 software to assess the quality of evidence for the meta-analysis results, evaluating each study based on research design type, risk of bias, inconsistency, indirectness, and imprecision. The evidence quality is classified into three levels: high, moderate, and low. To ensure the objectivity and reliability of the assessment, two researchers independently performed the scoring.

### 2.4 Data extraction

In this study, we conducted a comprehensive analysis of both qualitative and quantitative data on the relationship between childhood overweight/obesity and puberty development indicators in boys. Meta-analysis was performed to estimate the combined effect when two or more studies reported similar effect measurements for the same outcome. The primary outcomes included early testicular enlargement (testicular volume reaching Tanner Stage 2, ≥4 ml) and the age at early testicular enlargement. Secondary outcomes included the impact of waist circumference and body fat percentage on early testicular enlargement, the age of pubic hair development, and the age of peak height velocity (APHV).

Two researchers independently extracted the data based on the inclusion and exclusion criteria, resolving any discrepancies through discussion with other researchers. The data extracted included: 1.Basic characteristics of the included studies: first author, publication date, study type, region, sample size, age, and follow-up frequency. 2.Outcome indicators: early testicular enlargement, the age of early testicular enlargement, waist circumference, body fat percentage, the appearance of pubic hair, and peak height velocity.

### 2.5 Statistical analysis

Risk ratios (RR) and 95% confidence intervals (CI) were used as effect sizes to assess outcomes, with RR representing the effect measure in the analysis. Hazard ratios (HR) and odds ratios (OR) were treated as RR in the analysis. To avoid potential bias, the exact statistical methods for converting HR and OR to RR are as follows: for low event rates, log(HR) ≈ log(RR), and log(OR) ≈ log(RR). Some studies provided only the mean age and standard deviation for each puberty milestone under different weight statuses. In such cases, we calculated the mean difference and standard deviation between the two groups before performing the meta-analysis. The chi-square test was used to assess heterogeneity among the included studies. A P-value < 0.05 was considered indicative of statistical heterogeneity, and the I² statistic was used to measure the degree of heterogeneity. I² values of < 25%, 25-50%, and > 50% were considered to represent low, moderate, and high heterogeneity, respectively (17). If no significant heterogeneity was found, the fixed-effect model (Mantel-Haenszel) was used for data pooling; if significant heterogeneity was present, the random-effects model was applied, and subgroup analyses were performed to explore potential sources of heterogeneity. Sensitivity analysis was also conducted to assess the influence of individual studies on the results. To evaluate potential publication bias, funnel plots and Egger’s test were used, with a P-value < 0.05 considered statistically significant. Due to the limited number of studies on other outcomes, publication bias, sensitivity analysis, and subgroup analysis were only tested for the meta-analysis on early testicular enlargement.

All analyses, except for Egger’s test and sensitivity analysis, were conducted using Review Manager (version 5.4). Egger’s test and sensitivity analysis were performed using STATA (version 17.0, Computer Resource Center, USA).

## 3 Results

### 3.1 Literature search strategy and baseline characteristics

**Figure 1:**
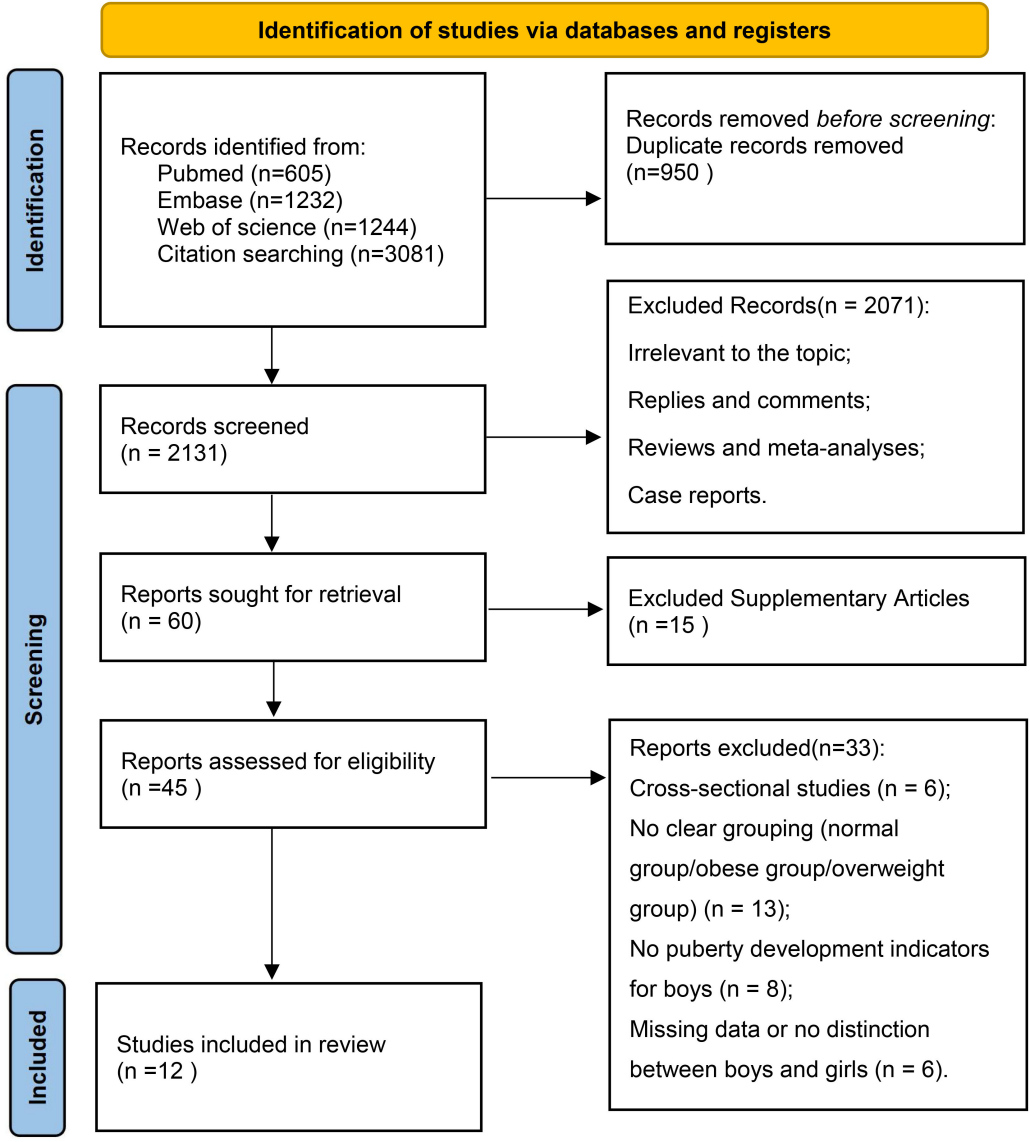
Literature search flowchart.

A search of selected public databases yielded 3,081 studies, which were screened, excluded, and quality-assessed based on the established inclusion and exclusion criteria. Ultimately, 12 cohort studies were included in this meta-analysis (10, 18–28). All included studies were prospective cohort studies conducted in English. The specific search process is shown in **Figure 1**, based on the inclusion and exclusion criteria. **Table 1** summarizes the baseline characteristics of the included studies. According to the Newcastle-Ottawa Scale (NOS) scores, 11 of the studies were rated as high quality, and 1 study was rated as moderate quality.

**Table 1:** Characteristics of Studies Included in the Systematic Review.

Author	Country	Sample Size	Age	Follow-up Frequency	Outcome Variables	NOS
**Deardorff 2022**	USA	NW:64; OW:23; OB:49	5.0	7 assessments: 9–14 years	Testicular enlargement and pubic hair	High
**Aghaee 2022**	USA	NW:46627; OW:9815; OB:9624	5.0–6.0	≥6 years, electronic medical records (2010–2021)	Testicular enlargement and pubic hair	High
**Li 2022**	China	645	9.1 (±0.6)	Every 6 months from 2017 to 2020	Testicular enlargement	High
**Fang 2022**	China	NW:317; OW/OB:62	9.03 (±0.98)	Annually	Testicular enlargement	High
**Chen 2021**	USA	NW:422; OW:115; OB:126	2.0–7.0	Pediatric follow-up until age 21	Peak height velocity age	High
**Pereira 2021**	Chile	NW:948; OW/OB:207	4.0	Every 6 months starting at age 7	Testicular enlargement	High
**Brix 2020**	Denmark	NW:4588; OW:494; OB:407	7.1 (7.0–7.2)	Every 6 months starting at age 11.5 until age 18 or full maturity	Testicular volume reaching Tanner Stage 2, pubic hair development	High
**Busch 2020**	Denmark	NW:536; OW:124; OB:218	4.2–17.0	Assessments at enrollment and 1 year after enrollment	Testicular volume reaching Tanner Stage 2 and pubic hair development	moderate
**Chen 2019**	China	3109	10.0–12.0	assessments from age 11, 12 to 18	Testicular enlargement	High
**Li 2018**	China	537; OW:79; OB:68	8.59 (±1.20)	7 follow-ups (every 6 months)	Testicular enlargement	High
**Leonibus 2013**	Italy	NW: 27 OB: 44	9.07 (±0.52)	Every 6 months until adult height is reached	Testicular volume reaching Tanner Stage 2 and peak height velocity	High
**Lee 2025**	USA	221764	8.43±0.69	7 assessments in national health insurance data (2008-2020)	Testicular enlargement and pubic hair	High

Note: Testicular enlargement is defined as the testicular volume reaching Tanner Stage 2; pubic hair is defined as the appearance of pubic hair. Abbreviations: AO, abdominal obesity; NOS, Newcastle-Ottawa Scale; NW, normal weight; OB, obesity; OW, overweight (excluding obesity). Bold values indicate the first author and year of publication.

### 3.2 Early testicular enlargement

#### 3.2.1 Risk of early testicular enlargement

**Figure 2:**
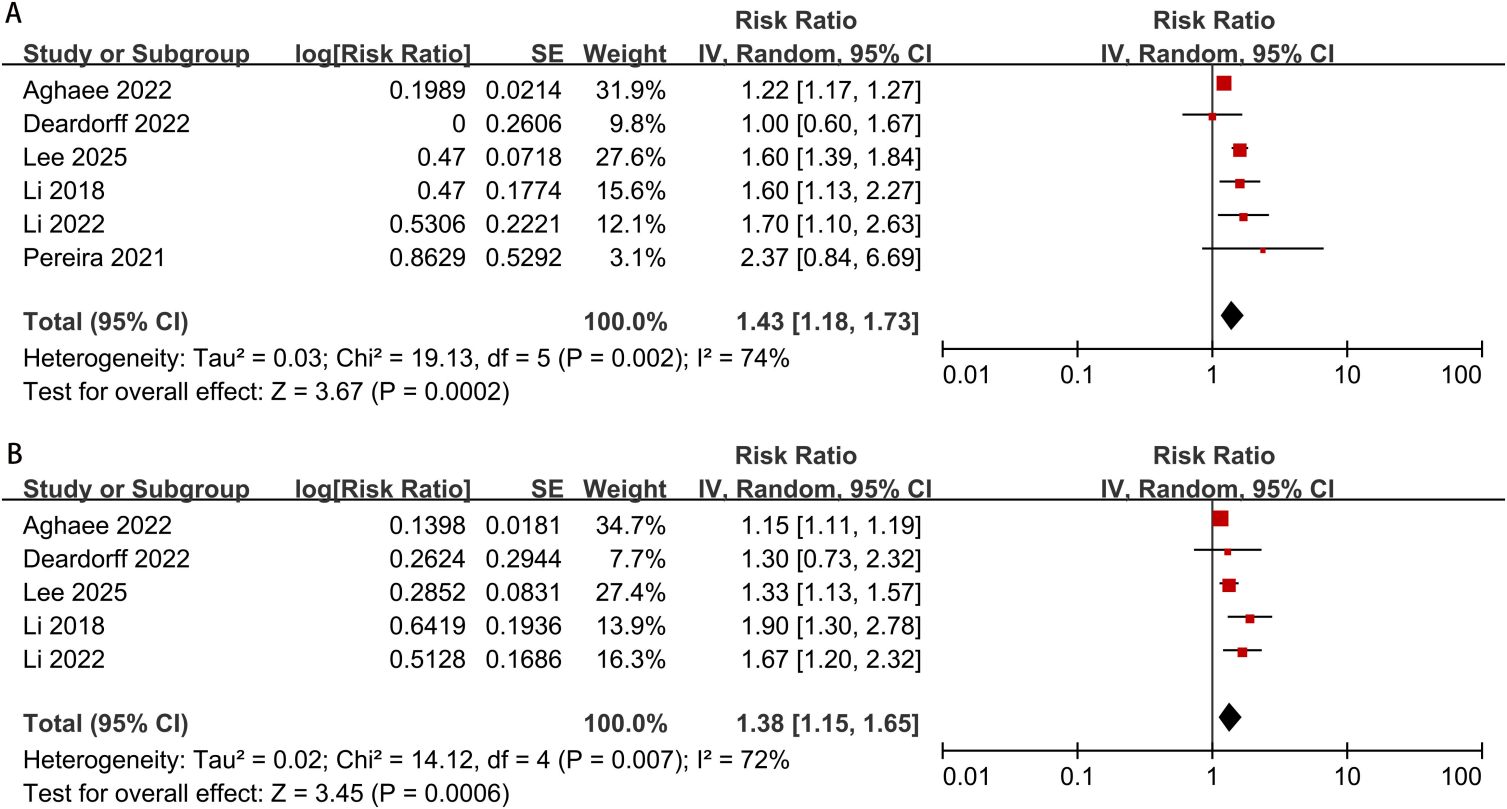
Meta-analysis forest plot of early testicular enlargement in obese **(A)** or overweight **(B)** boys during puberty.

**Figure 3:**
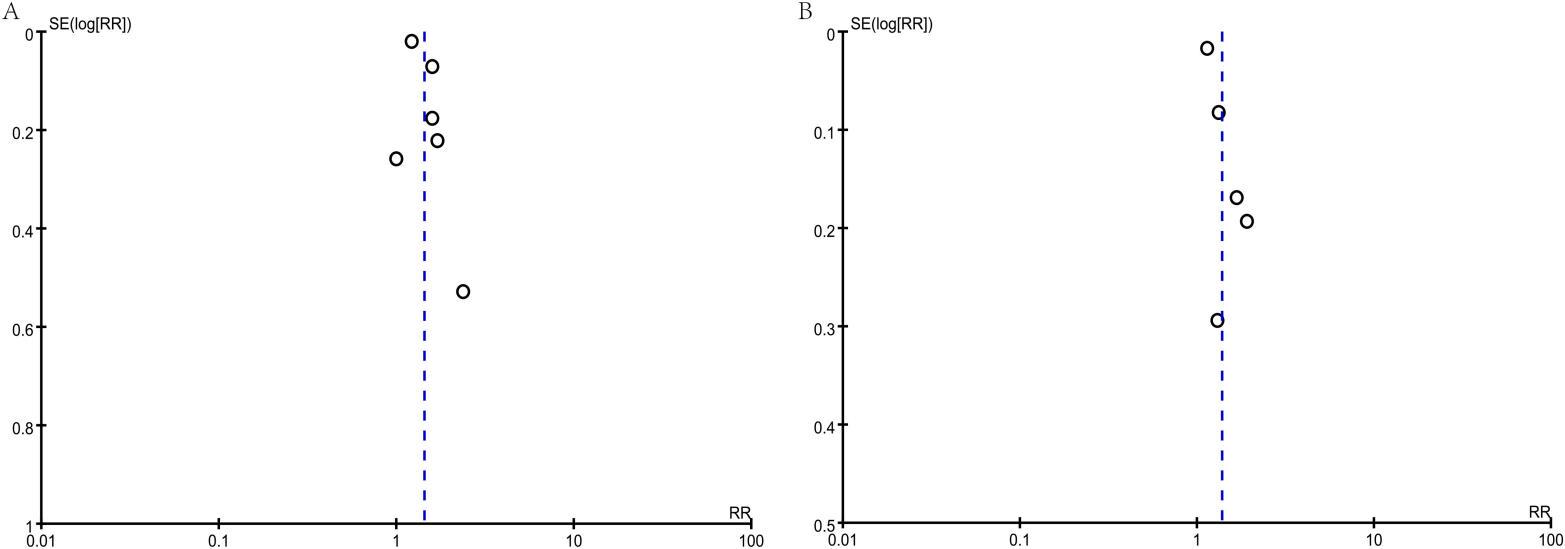
Meta-analysis funnel plot of early testicular enlargement in obese **(A)** or overweight **(B)** boys during puberty.

**Figure 4:**
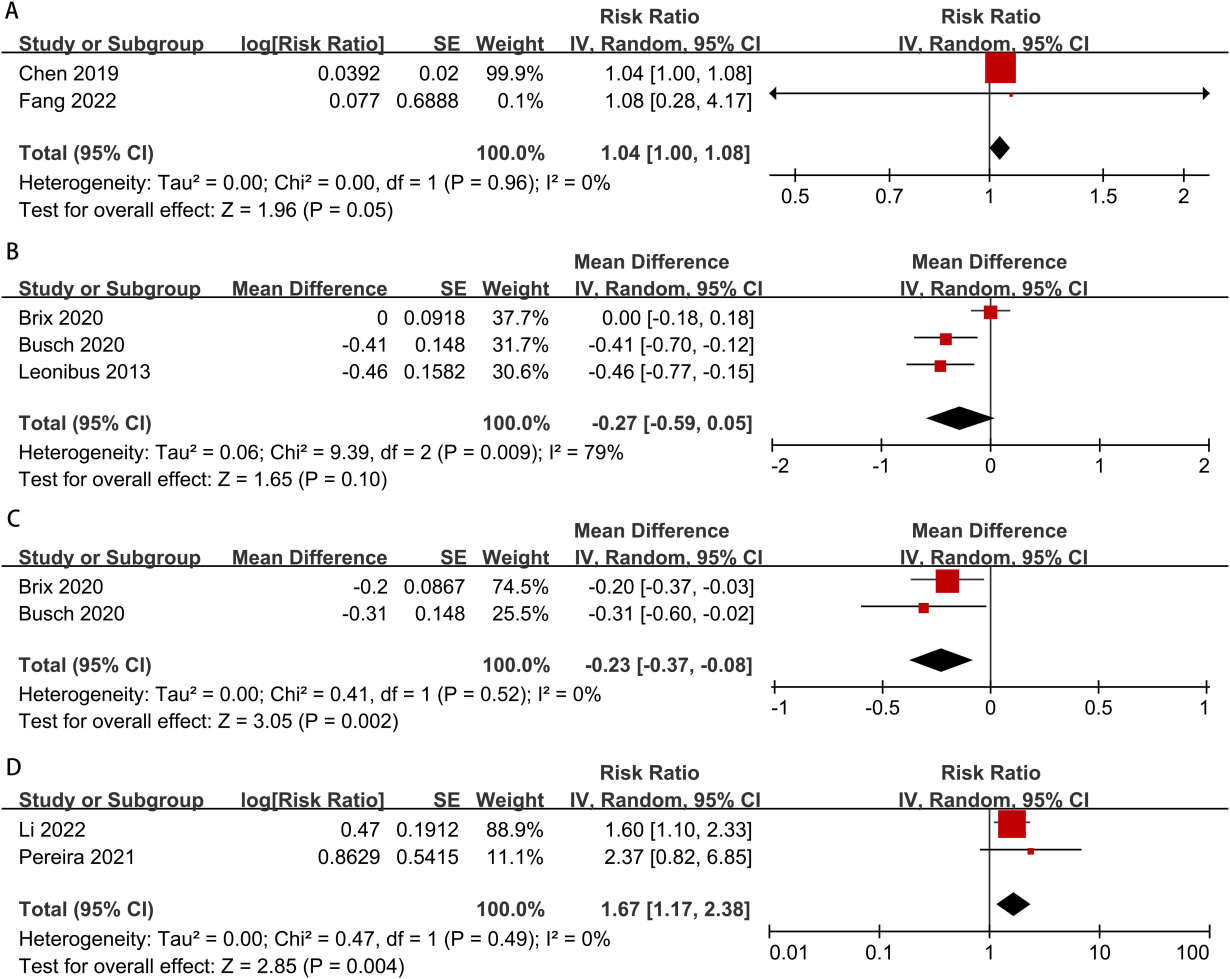
Meta-analysis forest plot of factors associated with early testicular enlargement in boys during puberty (**(A)** combined overweight and obesity, **(B)** age of testicular enlargement in obese boys, **(C)** age of testicular enlargement in overweight boys, **(D)** waist circumference and body fat percentage).

The meta-analysis on the risk of early testicular enlargement included 6 studies and found that overweight and obese boys had a higher risk of early testicular enlargement compared to normal-weight boys. For overweight boys, the risk ratio (RR) was 1.38 (95% CI, 1.15–1.65; I² = 72%, p = 0.0006), and for obese boys, the RR was 1.43 (95% CI, 1.18–1.73; I² = 74%, p = 0.0002) (**Figure 2**). Egger’s test and funnel plots indicated no publication bias (overweight: p = 0.186; obese: p = 0.143) (**Figure 3**). Subgroup analysis, as shown in **Table 2**, revealed regional differences in the association between overweight and early testicular enlargement. In the U.S., the RR (95% CI) for overweight boys was 1.11 (1.03–1.20), while in the Asia-Pacific region, the RR (95% CI) for overweight was 1.40 (1.22–1.60) (I² for subgroup difference = 88.4%; p for subgroup difference = 0.003). For obesity, no regional differences in effect estimates were observed (I² for subgroup difference = 71.4%; p for subgroup difference = 0.06). Additionally, we performed a subgroup analysis based on the baseline age at which the children’s weight was measured (≤6 years vs. >6 years). Among children with weight measurements taken after the age of 6, the association between overweight/obesity and early testicular enlargement was stronger. For instance, in children aged 6 years or younger, the RR (95% CI) for overweight was 1.15 (1.11–1.19), whereas for those older than 6 years, the RR (95% CI) for overweight was 1.53 (1.23–1.91) (I² for subgroup difference = 84.3%; p for subgroup difference = 0.01). Similar subgroup differences were observed for obese boys. Further subgroup analysis based on the sample size of the included studies showed significant differences between groups for overweight boys in terms of early testicular enlargement (I² for subgroup difference = 83.2%; p for subgroup difference = 0.01). Additionally, two studies combined overweight and obese boys into one group. Compared to normal-weight boys, the results showed that overweight or obese boys had a higher risk of early testicular enlargement (combined RR = 1.04; 95% CI, 1.00–1.08; I² = 0%, p = 0.05) (**Figure 4A**).

**Table 2:** Subgroup Analysis of Early Testicular Enlargement.

	Early Testicular Enlargement					
	Obesity			Overweight		
	Study Quantity	RR[95%CI]	P	Study Quantity	RR[95%CI]	P
**Region**			**0.06**			0.003*
Asia	4	1.47 [1.23, 1.76]	<0.0001	4	1.40 [1.22, 1.60]	<0.00001
USA	3	1.19 [1.04, 1.36]	0.009	2	1.11 [1.03, 1.20]	0.005
**Age of Weight Measurement**			<0.0001*			0.01*
≤6 years	3	1.22 [1.18, 1.26]	<0.00001	2	1.15 [1.11, 1.19]	<0.00001
>6 years	4	1.62 [1.43, 1.83]	<0.00001	3	1.53 [1.23, 1.91]	0.0001
**Sample Size**			**0.68**			**0.01**
Sample Size ≤5000	4	1.50 11.15,1.96]	0.003	3	1.68 [1.34, 2.12]	<0.00001
Sample Size >5000	2	1.39 [1.06, 1.81]	0.02	2	1.21 [1.06, 1.38]	0.006

Bold values indicate the subgroup categories and the p values from the statistical analyses.

#### 3.2.2 Age of early testicular enlargement

The meta-analysis results indicated that overweight boys experienced earlier testicular enlargement compared to normal-weight boys. However, no significant difference was observed in the obese group (overweight: MD = -0.23, 95% CI: -0.37 to -0.08, p = 0.002; obesity: MD = -0.23, 95% CI: -0.37 to 0.08, p = 0.10) (**Figures 4B, C**).

#### 3.2.3 Impact of waist circumference and body fat percentage on early testicular enlargement in boys

Two studies on abdominal obesity and the risk of testicular enlargement were included, and the results showed that boys with abdominal obesity had an increased risk of early testicular enlargement (RR = 1.67; 95% CI: 1.17–2.38; I² = 0%, p = 0.004) (**Figure 4D**).

### 3.3 Pubic hair development

**Figure 5:**
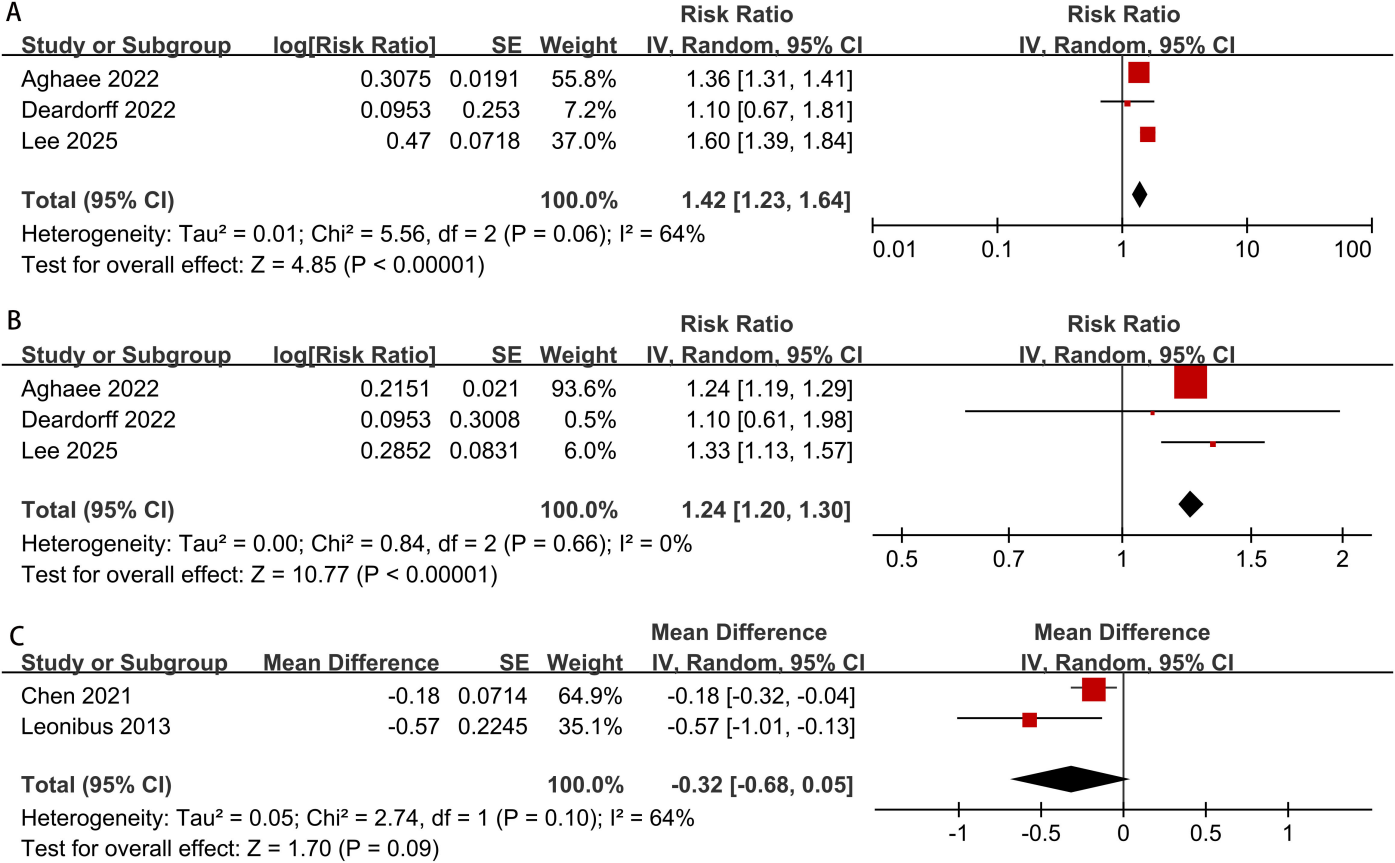
Meta-analysis forest plot of obesity and overweight in boys during puberty (**(A)** obesity and pubic hair development, **(B)** overweight and pubic hair development, **(C)** obesity and peak height velocity).

The meta-analysis results showed that overweight or obese boys had a higher risk of early pubic hair development compared to normal-weight boys (overweight: RR = 1.24; 95% CI: 1.20–1.30; I² = 0%, p < 0.00001; obesity: RR = 1.42; 95% CI: 1.23–1.64; I² = 0%, p < 0.00001) (**Figures 5A, B**).

### 3.4 Peak height velocity

The meta-analysis results revealed no significant statistical difference in peak height velocity between obese boys and normal-weight boys (MD = -0.32, 95% CI: -0.68 to 0.05, I² = 64%, p = 0.09) (**Figure 5C**).

### 3.5 Sensitivity analysis

**Figure 6:**
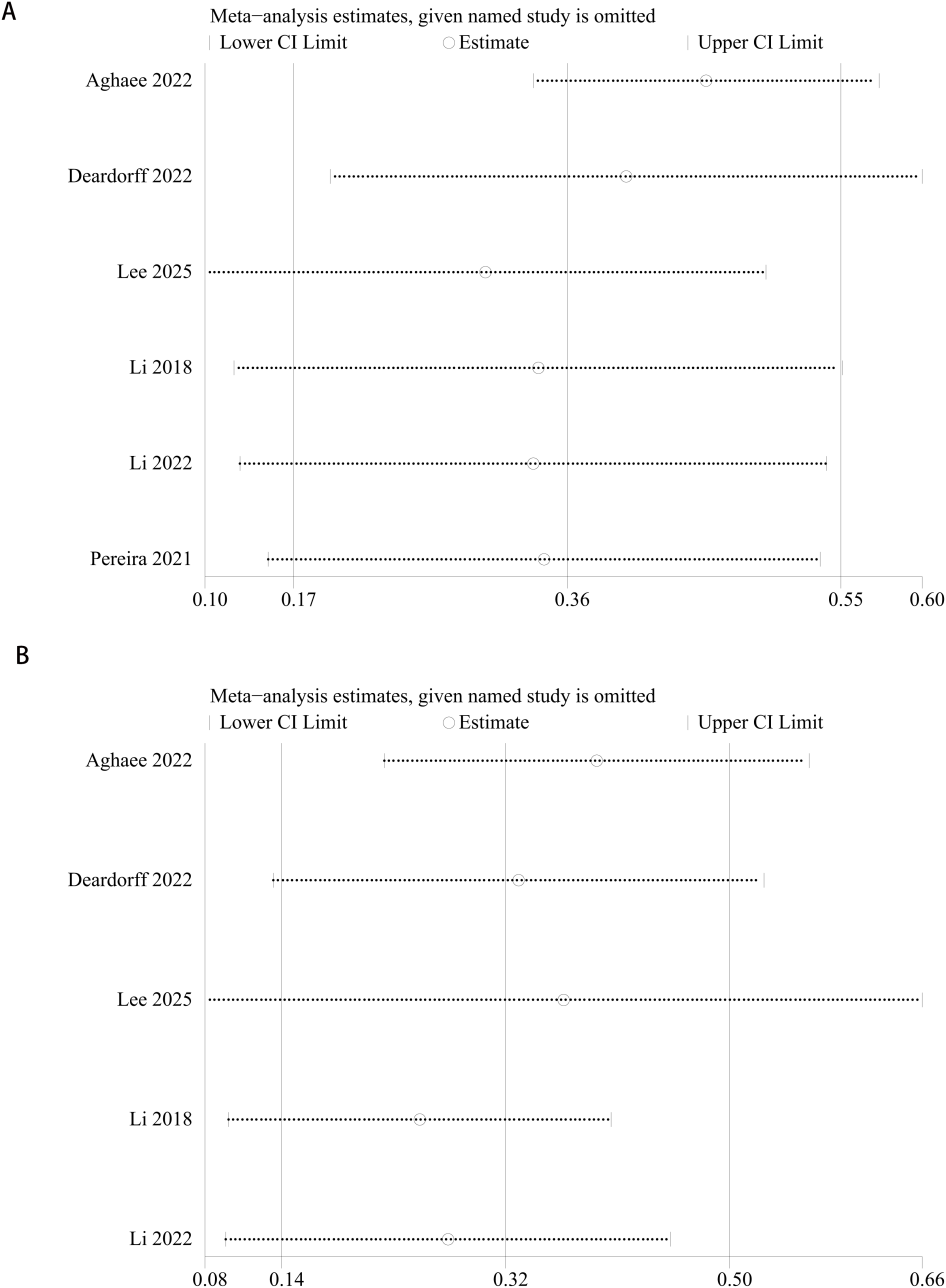
Sensitivity analysis of the meta-analysis on early testicular enlargement in obese **(A)** or overweight **(B)** boys during puberty.

A sensitivity analysis was performed on the risk of early testicular enlargement in overweight and obese boys by assessing the impact of excluding each study on the overall RR value. The results showed that excluding any individual study did not significantly change the RR value (p > 0.05), suggesting that our findings are robust and reliable. For details, see **Figure 6**.

### 3.6 Grade system

The GRADE system assessment indicated that the evidence quality for the risk of early testicular enlargement was high. However, the evidence quality for the age of testicular enlargement, pubic hair development, impact of waist circumference and body fat percentage on early testicular and peak height velocity was moderate, as shown in **Table 3**.

**Table 3:** Evidence Quality Assessment Results using the GRADEpro System.

Outcome	Number of Studies Included	Evidence Quality Assessment					Evidence Level
		Bias Risk	Inconsistency	Indirectness	Imprecision	Other Factors	
Risk of Early Testicular Enlargement	6	N	N	N	N	N	High
Age of Testicular Enlargement	3	S	N	N	N	N	moderate
Impact of Waist Circumference and Body Fat Percentage on Early Testicular Enlargement in Boys	2	S	N	N	N	N	moderate
Pubic Hair Development	3	S	N	N	N	N	moderate
Age of Peak Height Velocity	2	S	N	N	N	N	moderate

## 4 Discussion

This study systematically reviewed and summarized 12 cohort studies to assess the impact of overweight and obesity on male puberty development, focusing on key puberty events such as testicular enlargement, pubic hair development, and peak height velocity. We found that overweight and obese boys had a significantly higher risk of early testicular enlargement compared to normal-weight boys (overweight: RR 1.38; 95% CI, 1.15–1.65; I² = 72%, p = 0.0006; obesity: RR 1.43; 95% CI, 1.18–1.73; I² = 74%, p = 0.0002). This conclusion was confirmed through multiple subgroup analyses, particularly for children over the age of 6, where the association between overweight and early testicular enlargement was more pronounced (RR [95% CI] = 1.53 [1.23–1.91]). Additionally, overweight boys showed earlier pubic hair development compared to normal-weight boys.

The outcome measures used to assess male puberty development were not consistent across studies, with most evaluating development based on the age of testicular enlargement. In addition to testicular enlargement, other indicators such as the age of testicular enlargement, pubic hair appearance, peak height velocity, first ejaculation, and voice changes were also considered (29). However, due to the limited number of studies addressing these outcomes, meta-analysis could not be performed for all factors. The study by Li et al. found that a higher prepubertal body mass index in boys was significantly associated with the timing of first ejaculation (HR: 1.054, 95% CI: 1.004–1.106) and testicular development (HR: 1.098, 95% CI: 1.063–1.135) (25). Another cohort study found that, among 5,489 Danish boys, the first ejaculation occurred 0.13 years earlier in overweight boys and 0.23 years earlier in obese boys compared to normal-weight boys. Furthermore, a cohort study involving 1,434 Chinese boys found that overweight/obese boys had a higher risk of early voice changes compared to normal-weight boys (OR: 1.05; 95% CI: 1.02–1.08; P < 0.05) (10).

Research on the impact of obesity and overweight on girls’ puberty development is relatively consistent, with a general consensus that obesity leads to early puberty in girls. Numerous studies have shown that overweight or obese girls experience menarche and breast development earlier than their normal-weight counterparts, and this association has been validated across multiple races and regions (30). For instance, a study conducted in the U.S. and Europe found that for every one standard deviation increase in girls’ BMI, the age of menarche would advance by approximately 4.5 months (23). Other research has suggested that higher body fat levels in girls during early puberty, along with changes in hormones such as leptin and insulin, may play a key role in driving early sexual maturation (31).

However, research on the impact of obesity on male puberty development is more limited and somewhat controversial. Some studies suggest that obesity does not significantly affect male puberty development. For example, a previous study by Li et al. found that obesity did not significantly advance the age of testicular enlargement or the onset of first ejaculation in boys (13). Additionally, research by Thomas Reinehr and colleagues did not confirm that obese boys enter puberty earlier than normal-weight boys. The researchers proposed that the onset of puberty in boys is influenced by a broader range of endocrine factors, not just weight (32, 33). Despite these findings, an increasing number of studies suggest that obesity also affects male puberty development, leading to early sexual maturation. A large cohort study found that obese boys experience earlier testicular enlargement and first ejaculation compared to normal-weight boys (27). Another study indicated that overweight and obese boys reach earlier puberty milestones, such as testicular development and body hair growth, with obese boys being at a higher risk for early puberty (RR = 1.90; 95% CI [1.30–2.78]) (19). These studies suggest that obesity leads to hormonal changes, particularly an imbalance between androgens and estrogens, which may be a key factor driving early sexual maturation in boys.

Puberty is a critical developmental phase for boys, bringing about significant physical, hormonal, and psychological changes (34, 35). During this period, the body undergoes rapid growth and maturation, including the enlargement of reproductive organs, the development of secondary sexual characteristics such as pubic hair, and an increase in muscle mass. Hormonal changes, particularly the secretion of gonadotropin-releasing hormone (GnRH), testosterone, and growth hormone, play a central role in driving these changes (36, 37). Research has shown that obesity disrupts the hormonal signals regulating puberty, thereby influencing male development and accelerating the onset of puberty. In addition to hormonal imbalances, obesity leads to insulin resistance and associated metabolic disturbances, which further affect the timing of puberty and the development of secondary sexual characteristics (38).

The interaction between obesity and puberty is crucial for understanding male development, as both factors seem to amplify each other’s effects. Puberty, by increasing hormonal activity, may exacerbate the metabolic disruptions caused by obesity, creating a feedback loop that accelerates physical and hormonal changes. This cyclical interaction underscores the complexity of the relationship between obesity and puberty in boys (39). Existing studies have proposed several potential mechanisms for the relationship between obesity/overweight and early puberty development in boys. One key mechanism is that obesity may accelerate the onset of puberty by increasing the activity of aromatase, which promotes the conversion of androgens to estrogens. Aromatase expression is higher in fat tissue, and the elevated estrogen levels in obese children may lead to early activation of the gonadal axis, thereby prompting early puberty onset. Related studies have shown that obese children are exposed to higher levels of estrogen in their tissues, which may be a key factor in the early onset of puberty in these children (40). Additionally, obesity may promote early puberty by influencing the secretion and sensitivity of hormones in the body. In particular, leptin levels are typically higher in obese children compared to normal-weight children. Leptin is an important endocrine signaling molecule that regulates energy balance and influences reproductive system development. As body weight increases, leptin levels gradually rise, which may be a critical factor in the early onset of puberty in obese children (41). Leptin acts on the hypothalamus to stimulate the secretion of kisspeptin, which in turn activates the hypothalamic-pituitary-gonadal (HPG) axis, promoting the secretion of gonadotropin-releasing hormone (GnRH) and ultimately triggering the synthesis and secretion of sex hormones, thus driving the progression of puberty.

Moreover, obese children often experience insulin resistance, leading to elevated insulin levels, which may further disrupt hormone balance. Studies have shown that the interaction between insulin and sex hormones significantly impacts the onset of puberty. Elevated insulin levels may promote early sexual maturation by affecting the synthesis of estrogens and androgens or by directly acting on the gonads (42). While existing research has proposed biological mechanisms for the association between obesity/overweight and early puberty in boys, many unknowns remain. Future studies should explore these mechanisms through larger sample sizes and more rigorous experimental designs and investigate whether these mechanisms differ from those in girls.

This study has several limitations. First, all the included studies were observational, meaning causality cannot be determined, and the underlying mechanisms remain unclear. Second, differences in sample sizes and regions may introduce heterogeneity in the results, as most studies focused on specific regions, which could limit the generalizability of the findings. Furthermore, due to the limited number of studies included, the statistical power of the funnel plot and Egger test for assessing publication bias may be insufficient, and the results of the subgroup analysis and certain outcomes are only suggestive. Lastly, the definition and assessment methods for the onset of puberty varied across studies. Indicators such as first ejaculation, voice changes, and puberty development in boys with different BMI trajectories lacked sufficient data for meta-analysis. Future research should employ more precise measurement tools and standardized methods to validate these findings. The strength of this study lies in its systematic review and meta-analysis methodology, which synthesizes data from several high-quality cohort studies and provides robust evidence for the relationship between obesity/overweight and early puberty development in boys. Unlike previous studies, this study specifically focuses on the male population, addressing a significant research gap. Additionally, the use of strict inclusion criteria and various analytical methods enhances the reliability and generalizability of the results. By analyzing multiple key puberty milestones, we were able to offer a comprehensive assessment of the impact of obesity on puberty.

## 5 Conclusion

This systematic review and meta-analysis shows a significant association between overweight/obesity and early puberty development in boys, particularly with regard to testicular enlargement, pubic hair development, and the impact of waist circumference and body fat percentage on early testicular enlargement. Compared to normal-weight boys, overweight and obese boys have a significantly higher risk of early testicular enlargement. Subgroup analysis suggests that this association is more pronounced in children older than 6 years. Although no significant difference in peak height velocity was observed between obese and normal-weight boys, overweight and obesity still significantly affect male puberty development. Given the limited number of studies included in this meta-analysis, future research should validate these findings through large-scale cohort studies with more rigorous methodologies.

## Data Availability

The original contributions presented in the study are included in the article/supplementary material. Further inquiries can be directed to the corresponding author.
